# The contribution of maternal characteristics and cesarean delivery to an increasing trend of severe maternal morbidity

**DOI:** 10.1186/s12884-018-2169-3

**Published:** 2019-01-09

**Authors:** Stephanie A. Leonard, Elliott K. Main, Suzan L. Carmichael

**Affiliations:** 1 Division of Neonatal and Developmental Medicine, Department of Pediatrics, Stanford University School of Medicine, Stanford, California, USA; 2 Center for Population Health Sciences, Stanford University School of Medicine, Stanford, California, USA; 3 Department of Obstetrics and Gynecology, Stanford University School of Medicine, California, USA; 4 California Maternal Quality Care Collaborative, California, USA

**Keywords:** Cesarean section, Comorbidity, Maternal age, Maternal health, Obesity, Pregnancy complications, Severe maternal morbidity

## Abstract

**Background:**

Severe maternal morbidity – life-threatening childbirth complications – has more than doubled in the United States over the past 15 years, affecting more than 50,000 women (1.4% of deliveries) annually. During this time period, maternal age, obesity, comorbidities, and cesarean delivery also increased and may be related to the rise in severe maternal morbidity. We sought to evaluate: (1) the association of advanced maternal age, pre-pregnancy obesity, pre-pregnancy comorbidities, and cesarean delivery with severe maternal morbidity, and (2) whether changes in the prevalence of these risk factors affected the trend of severe maternal morbidity.

**Methods:**

This population-based cohort study used linked birth record and patient discharge data from live births in California during 2007–2014 (*n* = 3,556,206). We used multivariable logistic regression models to assess the association of advanced maternal age (≥35 years), pre-pregnancy obesity (body mass index ≥30 kg/m^2^), pre-pregnancy comorbidity (index of 12 conditions), and cesarean delivery with severe maternal morbidity prevalence and trends. Severe maternal morbidity was identified by an index of 18 diagnosis and procedure indicators. We estimated odds ratios, predicted prevalence, and population attributable risk percentages.

**Results:**

The prevalence of severe maternal morbidity increased by 65% during 2007–2014. Advanced maternal age, pre-pregnancy obesity, and pre-pregnancy comorbidity also increased during this period, but cesarean delivery did not. None of these risk factors affected the increasing trend of severe maternal morbidity. However, the pre-pregnancy risk factors together were estimated to contribute to 13% (95% confidence interval: 12, 14%) of severe maternal morbidity cases in the study population overall, and cesarean delivery was estimated to contribute to 37% (95% confidence interval: 36, 38%) of cases.

**Conclusions:**

Pre-pregnancy health and cesarean delivery are important risk factors for severe maternal morbidity but do not explain an increasing trend of severe maternal morbidity in California during 2007–2014. Investigation of other potential contributors is needed in order to identify ways to reverse the trend of severe maternal morbidity.

**Electronic supplementary material:**

The online version of this article (10.1186/s12884-018-2169-3) contains supplementary material, which is available to authorized users.

## Background

The United States has the highest maternal mortality rate of all developed countries, and it is the only one with an increasing rate [1]. For every death, an additional 50 to 100 women experience life-threatening complications – affecting more than 50,000 women each year [2]. The Centers for Disease Control and Prevention (CDC) identify cases of severe maternal morbidity using an index of 18 indicators of a significant event or condition at delivery, such as blood transfusion, renal failure, and respiratory distress [3]. These complications can be life threatening, have long-term effects on a woman’s health, and adversely affect her infant’s health and well-being [2, 4–6]. According to the CDC, the annual U.S. prevalence of severe maternal morbidity has more than doubled over the past 15 years – from 0.6% of deliveries in 1998 to 1.4% of deliveries in 2014 [3].

In recent decades, the characteristics of women giving birth have significantly changed. Advanced maternal age, obesity, and comorbidities (such as hypertension and diabetes) have all increased in prevalence [7–11]. Cesarean delivery also became more common – rising from approximately 1 in 5 births in 1996 to 1 in 3 births by 2006 [12]. These factors have all been associated with a higher risk of severe maternal morbidity [6, 13–22]. It has been widely postulated that increases in these risk factors explain worsening trends in maternal health [2–4, 23, 24]. Previously, Kuklina et al. [5] found that only cesarean delivery, but not maternal age or comorbidities (obesity was not considered), was associated with an increasing trend of severe delivery complications in the U.S. from 1998 to 2005. The national cesarean delivery rate steadily increased from 1996 to 2009, but then plateaued and in 2013 began to decrease [25]. To our knowledge, the contribution of trends in maternal health characteristics and cesarean delivery to trends in maternal health in recent years has not been studied. Understanding how these factors have affected recent trends in severe maternal morbidity is needed to inform public health interventions aimed at improving maternal health. To fill this evidence gap, we evaluated the association of advanced maternal age, pre-pregnancy obesity, pre-pregnancy comorbidities, and cesarean delivery with severe maternal morbidity, and whether changes in the prevalence of these risk factors affected the trend of severe maternal morbidity from 2007 to 2014 in California, where 1 in 8 U.S. births occurs.

## Methods

This cohort study used data from 4,063,106 recorded live births that occurred in California from January 1, 2007 through December 31, 2014. California adopted the revised U.S. birth certificate in 2007, which added the collection of maternal weight and height information. The International Classification of Disease, 10th Edition, Clinical Modification was implemented in 2015. We restricted to delivery years in which the International Classification of Disease, 9th Edition, Clinical Modification (ICD-9-CM) was used for consistency in hospital coding practices. Birth certificate data were obtained from the California Department of Public Health (2007–2011) and the California Maternal Quality Care Collaborative (2012–2014). For 99% of deliveries, birth certificate data were previously linked to maternal delivery hospitalization discharge data collected by the Office of Statewide Health Planning and Development. If twins or other multiples were delivered, the maternal discharge data were duplicated in separate delivery records for each infant. We therefore selected the first delivery record if multiples were delivered. The final sample of complete cases included 3,556,206 deliveries with linked records and plausible gestational duration (> 20 weeks). We assessed differences between included and excluded subjects.

The outcome under study was severe maternal morbidity that occurred during the delivery hospitalization. We identified deliveries with severe maternal morbidity using ICD-9-CM diagnosis and procedure codes corresponding to 18 indicators (Additional file 1: Table S1) [3]. The CDC, along with its partners, created the list of indicators and ICD-9-CM codes to identify severe maternal morbidity when using administrative hospital discharge data.

The risk factors of interest were advanced maternal age, pre-pregnancy obesity, pre-pregnancy comorbidity, and cesarean delivery. Maternal age at delivery was collected on the birth certificate, and advanced maternal age was defined as ≥35 years. Pre-pregnancy body mass index (BMI) was calculated from self-reported pre-pregnancy weight and height on the birth certificate and maternal obesity was defined as BMI ≥30 kg/m^2^. Pre-pregnancy comorbidity was a binary variable defined as indication of any of the 12 pre-pregnancy medical conditions included in the obstetric comorbidity index created by Bateman et al. [26], reported either in the hospitalization record or on the birth certificate. These conditions included pulmonary hypertension, sickle cell disease, chronic renal disease, preexisting hypertension, chronic ischemic heart disease, congenital heart disease, systemic lupus erythematosus, human immunodeficiency virus, cardiac valvular disease, chronic congestive heart failure, asthma, and preexisting diabetes mellitus. Cesarean delivery was reported either in the hospitalization record (ICD-9-CM procedure code 74) or on the birth certificate. In descriptive analyses, we additionally categorized maternal age as < 20, 20–24, 25–29, 30–34, 35–39, or ≥ 40 years; pre-pregnancy BMI (kg/m^2^) as < 18.5 (underweight), 18.5–24.9 (normal weight), 25–29.9 (overweight), 30–34.5 (obesity class 1), 35–39.9 (obesity class 2), and ≥ 40 (obesity class 3); pre-pregnancy comorbidity as hypertension, diabetes, asthma, or other; and delivery method as vaginal without induction, vaginal with induction, primary cesarean without induction, primary cesarean with induction, and repeat cesarean. We used ICD-9-CM procedure codes 73.1, 73.4, 73.01 in delivery hospitalization records to identify labor induction, as previously validated [27]. Prior cesarean delivery was identified using birth certificate data and ICD-9-CM diagnosis code 654.2x in delivery hospitalization records. Confounding factors included in all multivariable regression models were selected a priori based on prior knowledge, directed acyclic graphs, and variables available in the dataset [2, 5, 6, 14, 17, 22]. These factors were reported on the birth certificate and included maternal race/ethnicity (U.S.-born Hispanic/Latina, foreign-born Hispanic/Latina, non-Hispanic White, Asian/Pacific Islander, non-Hispanic Black, Other), educational attainment (less than high school degree, high school degree or equivalent, some college, college degree), expected method of payment for delivery (private insurance or other), obstetric history (nulliparous, multiparous without previous cesarean delivery, multiparous with previous cesarean delivery), and twin/multiple birth. We additionally adjusted cesarean delivery models for placental conditions (abruption or previa; ICD-9-CM diagnosis code 641.x), preeclampsia (ICD-9-CM diagnosis codes 642.4, 642.5, 642.7), and gestational age because of their associations with cesarean delivery and severe maternal morbidity.

### Statistical analysis

We first assessed the distribution of maternal and delivery characteristics in women with and without severe maternal morbidity. We then tested the association of each risk factor of interest (advanced maternal age, pre-pregnancy obesity, pre-pregnancy comorbidity, and cesarean delivery) with severe maternal morbidity using multivariable logistic regression models, adjusted for race/ethnicity, education, payment method, obstetric history, and twin/multiple birth. Because of differences in temporality, which distinguish confounders and mediators, we additionally adjusted for advanced maternal age with obesity as the predictor; advanced maternal age and obesity with comorbidity as the predictor; and advanced maternal age, obesity, comorbidity, placental condition, preeclampsia, and gestational age with cesarean delivery as the predictor.

We then estimated population attributable risk percentages to understand the population-level implications of the associations between the risk factors of interest and severe maternal morbidity. Population attributable risk percentages account for both the strength of an association and the prevalence of the risk factor in the population. To calculate this measure, we used the multivariable logistic regression models to predict the prevalence of severe maternal morbidity if the risk factor of interest were eliminated. For example, severe maternal morbidity prevalence was predicted from the models after re-coding all cesarean deliveries to vaginal deliveries in the dataset (for heuristic purposes). We then calculated population attributable risk percentages as:

$$ 100\times \left(\frac{Observed\ prevalence- Predicted\ prevalence\ if\ risk\ factor\ eliminated}{Observed\ prevalence}\right) $$

We bootstrapped these simple substitution models 1000 times to obtain 95% confidence intervals.

We followed a similar approach to assess the effect of the risk factors of interest on the temporal trend of severe maternal morbidity. First, we calculated and plotted the annual prevalence of severe maternal morbidity and each risk factor. We then examined the trend in severe maternal morbidity using multivariable logistic regression models with delivery year as the independent variable. We sequentially adjusted the model for confounders and each risk factor of interest (advanced age, obesity, comorbidity, cesarean delivery) to determine their contributions to the trend. Finally, we calculated the predicted prevalence of severe maternal morbidity over time by stratifying the data by delivery year and modeling the association between each risk factor and severe maternal morbidity in each delivery year using multivariable regression models. This analytical step facilitates understanding the impact of the risk factors of interest on the population burden of severe maternal morbidity over time.

The CDC has reported that blood transfusions have driven the national increasing trend of severe maternal morbidity [3]. We therefore repeated all analyses for the outcome of severe maternal morbidity excluding cases for which blood transfusion was the only qualifying indicator (referred to below as ‘transfusion-only’ cases). All analyses were performed in R 3.4.2 and SAS 9.4.

## Results

Severe maternal morbidity occurred in 47,973 deliveries (1.35%). A higher proportion of people with severe maternal morbidity was ≥35 years old, obese, primiparous, lacked private health insurance, did not complete high school, and identified as non-Hispanic Black (Table 1). Pre-pregnancy comorbidities and cesarean delivery were two-fold higher in those with severe maternal morbidity (Table 1). Subjects excluded from analyses for missing or implausible data had a higher prevalence of severe maternal morbidity (1.59% vs. 1.35%), tended to have lower socioeconomic indicators, and delivered in earlier years than included subjects (Additional file 2: Table S2).

**Table 1:** Characteristics of deliveries with and without severe maternal morbidity, California, 2007–2014 (*n* = 3,556,205)

	Prevalence in deliveries with severe maternal morbidity (*n* = 47,973) No. (%)	Prevalence in deliveries without severe maternal morbidity (*n* = 3,508,232) No. (%)
Risk factors of interest – dichotomous		
Maternal age at delivery ≥35 years	11,173 (24)	639,568 (18)
Pre-pregnancy obesity	10,610 (22)	741,309 (21)
Pre-pregnancy comorbidity	6937 (15)	235,128 (7)
Cesarean delivery	29,532 (62)	1,143,410 (33)
Risk factors of interest – multiple categories		
Maternal age at delivery (years)		
< 20	4504 (9)	281,469 (8)
20–24	9396 (20)	731,140 (21)
25–29	11,077 (23)	939,816 (27)
30–34	11,823 (25)	916,240 (26)
35–39	8188 (17)	509,148 (15)
≥ 40	2985 (6)	130,420 (4)
Pre-pregnancy BMI group		
Underweight	2092 (4)	141,100 (4)
Normal weight	22,902 (48)	1,717,722 (49)
Overweight	12,369 (26)	907,102 (26)
Obesity Class 1	6111 (13)	446,573 (13)
Obesity Class 2	2660 (6)	185,634 (5)
Obesity Class 3	1839 (4)	109,102 (3)
Pre-pregnancy comorbidity		
Pre-pregnancy hypertension	2319 (5)	60,222 (2)
Pre-pregnancy diabetes mellitus	1225 (3)	39,299 (4)
Asthma	2613 (5)	125,423 (4)
Other comorbidity^1^	2116 (15)	26,159 (7)
Delivery method		
Vaginal without induction	14,190 (39)	1,955,468 (63)
Vaginal with induction	4251 (12)	409,355 (13)
Primary cesarean without induction	5977 (16)	169,941 (6)
Primary cesarean with induction	1366 (4)	37,317 (1)
Repeat cesarean	11,053 (30)	542,575 (17)
Sociodemographic factors		
Maternal education		
Did not complete high school	12,399 (26)	795,985 (23)
High school graduate or GED completed	12,659 (26)	914,409 (26)
Some college – no degree	11,335 (24)	867,550 (25)
College degree or higher	11,580 (24)	930,289 (27)
Private health insurance	21,016 (44)	1,672,011 (48)
Maternal race/ethnicity		
U.S.-born Hispanic/Latina	12,915 (27)	904,233 (26)
Foreign-born Hispanic/Latina	12,418 (26)	903,320 (26)
Non-Hispanic White	10,418 (22)	928,670 (26)
Asian/Pacific Islander	6105 (13)	438,649 (13)
Non-Hispanic Black	3852 (8)	176,510 (5)
Other	2265 (5)	156,851 (5)
Obstetric history		
Primiparous	21,134 (44)	1,391,190 (40)
Multiparous without prior cesarean	15,617 (33)	1,535,819 (44)
Multiparous with prior cesarean	11,222 (23)	581,224 (17)
Twin/multiple birth	54,189 (2)	3114 (6)
Preterm birth (< 37 weeks)	261,427 (7)	10,896 (23)
Placenta previa or abruption	5199 (11)	56,171 (2)
Preeclampsia	7116 (15)	116,198 (3)

BMI, body mass index (calculated as weight in kilograms divided by height in meters squared); GED, general educational development

^1^Other comorbidity includes pulmonary hypertension, sickle cell disease, chronic renal disease, chronic ischemic heart disease, congenital heart disease, systemic lupus erythematosus, human immunodeficiency virus, cardiac valvular disease, and chronic congestive heart failure [26]

Advanced maternal age and pre-pregnancy comorbidity were associated with severe maternal morbidity, but pre-pregnancy obesity was not (Table 2). Together, these pre-pregnancy factors were estimated to contribute to 13% (95% CI: 12, 14) of severe maternal morbidity cases in the study population. Cesarean delivery was associated with 2.7 times the risk of severe maternal morbidity (95% CI: 2.6, 2.7), compared to vaginal delivery, and was estimated to contribute to 37% (95% CI: 36, 38) of severe maternal morbidity cases in the population.

**Table 2:** Adjusted associations between risk factors of interest and severe maternal morbidity, California, 2007–2014, n = 3,556,206

Risk factor	Odds ratio (95% CI) for severe maternal morbidity	Population attributable risk percentage (95% CI) for severe maternal morbidity
Advanced maternal age	1.49 (1.45, 1.52)	7.5 (6.6, 8.4)
Pre-pregnancy obesity	0.99 (0.97, 1.01)	−0.2 (−1.2, 0.8)
Pre-pregnancy comorbidity	2.22 (2.16, 2.28)	7.8 (6.9, 8.6)
Cesarean delivery	2.66 (2.61, 2.73)	37.1 (36.0, 38.0)

*CI*, confidence interval

**Fig. 1:**
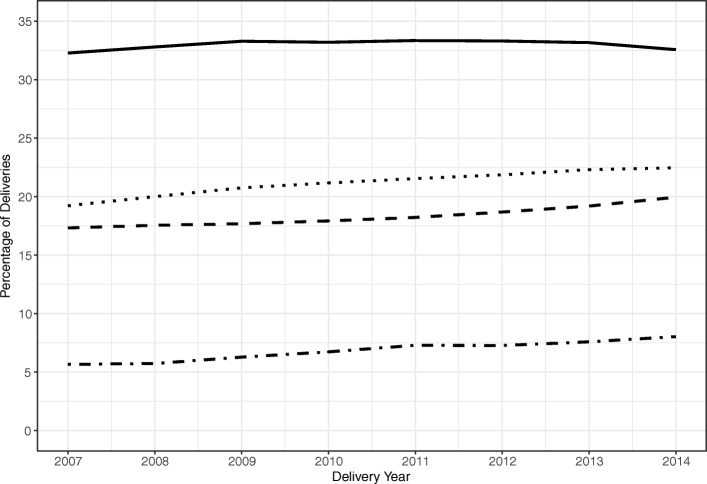
Prevalence of the risk factors of interest, 2007–2014. In descending order: cesarean delivery (—), pre-pregnancy obesity (· ·), advanced maternal age (− −), and pre-pregnancy comorbidity (· –)

The prevalence of severe maternal morbidity increased by 65% during the study period, from 1.02% in 2007 to 1.69% in 2014. Advanced maternal age, pre-pregnancy obesity, and pre-pregnancy comorbidity also increased in prevalence, but cesarean delivery did not (Fig. 1). Prevalence increased overall by 15% for advanced maternal age (from 17 to 20%), 17% for pre-pregnancy obesity (from 19 to 22%), and 42% for pre-pregnancy comorbidity (from 6 to 8%), whereas the prevalence of cesarean delivery remained relatively consistent (from 32.3 to 32.6%). These factors had negligible effects on the increasing trend of severe maternal morbidity; the odds of severe maternal morbidity increased by 7% (95% CI: 7, 8%) on average over each year from 2007 to 2014, which did not change after statistical adjustment for confounders and all risk factors of interest (Table 3).

**Table 3:** Odds ratios reflecting per-year change in severe maternal morbidity, with and without adjustment for selected risk factors, California, 2007–2014, *n* = 3,556,206

Regression model terms	Odds ratio for year (95% CI)
Year	1.07 (1.07, 1.08)
Year + sociodemographic & obstetric factors	1.07 (1.07, 1.08)
Year + sociodemographic & obstetric factors + advanced maternal age + obesity + comorbidity + cesarean delivery	1.07 (1.07, 1.08)

*CI*, confidence interval

**Fig. 2:**
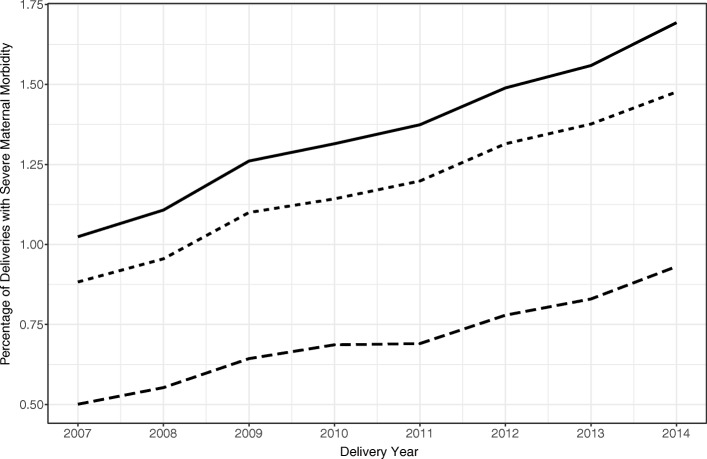
Observed and predicted annual prevalence of severe maternal morbidity, 2007–2014. Predictions were based on models that adjusted for confounders and set risk factors of interest to zero prevalence. In descending order: observed prevalence (−—), prevalence after setting advanced maternal age, obesity, and comorbidity to zero (· ·), and prevalence after additionally setting cesarean delivery to zero (− −)

When examining each delivery year separately, cesarean delivery had the largest impact on the predicted prevalence of severe maternal morbidity; advanced maternal age and pre-pregnancy comorbidity had similar impacts; and pre-pregnancy obesity had a negligible impact (Fig. 2; Additional file 3: Table S3). However, none of the risk factors substantially impacted the trend of severe maternal morbidity over time; the predicted annual rates of severe maternal morbidity were similar to the observed rates even after setting each risk factor to zero for everyone (Fig. 2; Additional file 3: Table S3).

Trend analysis results were similar after excluding blood transfusion-only cases. The prevalence of this more restrictive outcome increased by 57%, from 0.42% in 2007 to 0.64% in 2012, after which it largely leveled off (Additional file 4: Figure S1). After adjustment for risk factors, the average per-year increase from 2007 to 2012 was 8% (95% CI: 7, 9%).

## Discussion

In California, the prevalence of severe maternal morbidity increased 65% from 2007 to 2014. We found that advanced maternal age, pre-pregnancy comorbidity, and cesarean delivery, but not pre-pregnancy obesity, were associated with severe maternal morbidity. In addition, the prevalence of each of these risk factors increased from 2007 to 2014, except cesarean delivery. None of them, however, substantially explained the increasing trend of severe maternal morbidity. These findings do not support a widespread hypothesis that changes in women’s pre-pregnancy health, along with cesarean delivery, have driven an increase in adverse maternal health outcomes in the United States [2–4, 23, 24].

Compared to previous studies, we observed similar relative measures of association between severe maternal morbidity and maternal age ≥ 35 years [6, 13–17], pre-pregnancy comorbidities [14, 16, 18, 19], cesarean delivery [14, 15, 20], and sociodemographic and obstetric factors [6, 14–16, 20–22]. The association between pre-pregnancy obesity and severe maternal morbidity was not statistically significant, but has been moderate or null in other studies [17, 20]. Our study additionally estimated the population burden of severe maternal morbidity associated with these risk factors, which incorporates both relative risk and risk factor prevalence in the population. This information is useful for understanding the trend of severe maternal morbidity and designing interventions that are effective at the population level. These estimates suggested that cesarean delivery was associated with a much larger proportion of severe maternal morbidity than was any other risk factor. Cesarean delivery was associated with a 2.7-fold increased risk of severe maternal morbidity and a population attributable risk percentage of 37%, because of its relatively high prevalence of 33%. In comparison, pre-pregnancy comorbidity was associated with a 2.2-fold increase in severe maternal morbidity risk, but its population attributable risk percentage was 8% because of its relatively low prevalence of 6%.

Although cesarean delivery has a strong relationship with severe maternal morbidity, we did not find evidence that the increasing trend of severe maternal morbidity can be explained by cesarean delivery. In contrast, Kuklina et al. [5] found in a sample of deliveries in the U.S. during 1998–2005 that adjustment for cesarean delivery, but not maternal age or comorbidities, significantly attenuated increasing trends of severe obstetric complications. During that time period, cesarean delivery rates were increasing nationally and in California [5, 28]. Our current results show that the cesarean delivery rate in California began to stabilize in 2009 and decrease in 2013, which also occurred nationally [25]. This recent change of direction in the trend of cesarean delivery in California may explain why our results differ from those of Kuklina et al. [5] in an earlier time period; however, it is important to keep in mind that despite this stability in the prevalence of cesarean delivery, the prevalence of severe maternal morbidity continued to rise, and we still do not know why. It will be important to re-evaluate the contribution of cesarean delivery in the future, especially if the prevalence of cesarean delivery continues to decrease.

The results of our study were unexpected and underscore the need to study possible contributors to the trend of severe maternal morbidity beyond the factors that we examined. One such potential factor is changing patterns of availability of and access to care. For example, Hung et al. [29] found a complete loss of hospital obstetric services from 2004 to 2014 in 9% of rural counties in the United States. Counties with more vulnerable patient populations were also more likely to lose obstetric services than other rural counties. In addition, there is a complicated relationship between health care quality and severe maternal morbidity that deserves further consideration. In New York City, Howell et al. [16, 30] found that Hispanic and non-Hispanic black women were more likely to deliver at hospitals with higher severe maternal morbidity risk than white women, contributing to higher rates of severe maternal morbidity in these racial/ethnic minority groups. If marginalized obstetric patient populations in urban areas have increasingly sought care at hospitals with higher severe maternal morbidity risk, such a change could potentially contribute to an increasing trend of severe maternal morbidity.

There are important limitations in this study. The study was observational and relied on data collected for administrative and surveillance purposes, which could cause misclassification and precludes any causal conclusions. In particular, pre-pregnancy weight was self-reported, which could have introduced measurement error. A recent systematic review, however, concluded that error in self-reported pre-pregnancy weight largely did not bias associations with birth outcomes [31]. Available data limited the ability to study all variables of interest and may have resulted in residual confounding. The quantity and type of blood product used in a transfusion are unknown in our dataset and prevent direct identification of postpartum hemorrhage. The severe maternal morbidity measure used was created for administrative data and has been found to overestimate true positive severe maternal morbidity [32]; however, our results were similar after excluding the transfusion-only cases. This study was limited to the state of California and the results may not be generalizable to other regions, although it is noteworthy that 1 in 8 U.S. births occur in California, and it is a relatively diverse state with respect to race-ethnicity, socioeconomic factors, and geography.

This study also has several strengths. The study dataset was population-based, large, diverse, and contemporary. Linkage between vital records and patient discharge records enabled the study of variables not available in most administrative databases, such as the National Inpatient Sample and the Nationwide Readmissions Database. Severe maternal morbidity was measured using an index previously found to have reasonable validity as a population measure [32].

## Conclusions

Pre-pregnancy health and cesarean delivery are important risk factors for severe maternal morbidity but were not associated with an increasing trend of severe maternal morbidity in California during 2007–2014. Evaluation of potential contributors to trends in severe maternal morbidity provides evidence on which to base interventions and hopefully prevent serious complications during delivery. Although the vast majority of women do not experience such complications, the serious ramifications of and increasing trend in severe maternal morbidity warrant attention. This study suggests that efforts to understand trends in severe maternal morbidity should expand beyond advanced age, obesity, comorbidities, and cesarean delivery. However, the current downward trend of cesarean delivery suggests that public health efforts to reduce unnecessary cesarean deliveries are having some success. Women who deliver by cesarean are known to be at a much higher risk of severe maternal morbidity than women who deliver vaginally, and our findings suggest that efforts to avert unnecessary cesareans may hold promise for decreasing severe maternal morbidity prevalence in the future.

## Additional files

Additional file 1: **Table S1.** Severe maternal morbidity indicators and corresponding ICD-9-CM codes during delivery hospitalizations. These codes were used to identify the outcome studied. (DOCX 20 kb)

Additional file 2: **Table S2.** Comparison of characteristics for deliveries included in and excluded from the final study sample. Excluded subjects had a lower prevalence of the outcome, lower socioeconomic indicators, and delivered in earlier years. (DOCX 22 kb)

Additional file 3: **Table S3.** Observed and predicted severe maternal morbidity prevalence per 100 live births (95% confidence intervals), California, 2007–2014. The studied risk factors were related to the prevalence of SMM, but not its increase over time. (DOCX 21 kb)

Additional file 4: **Figure S1.** Annual prevalence of severe maternal morbidity excluding blood transfusion-only cases, California, 2007–2014. The prevalence increased from 2007 to 2012. (DOCX 66 kb)

## Abbreviations

CDC: Centers for Disease Control and Prevention
CI: confidence interval
