# Supplementary material for: The contribution of maternal characteristics and cesarean delivery to an increasing trend of severe maternal morbidity
Source: BMC Pregnancy Childbirth. 2019 Jan 9;19:16. doi: 10.1186/s12884-018-2169-3 (PMC6327483; doi:10.1186/s12884-018-2169-3)
Supplement: Supplementary file 1 — Table S1. Severe maternal morbidity indicators and corresponding ICD-9-CM codes during delivery hospitalizations. These codes were used to identify the outcome studied. (DOCX 20 kb) [file 12884_2018_2169_MOESM1_ESM.docx]

Additional file 1: **Table S1**. Severe maternal morbidity indicators and corresponding ICD-9-CM codes during delivery hospitalizations.

| **Severe Maternal Morbidity Indicator** | **Diagnosis (DX) or Procedure (PR)** | **ICD-9-CM** |
| --- | --- | --- |
| Acute myocardial infarction/Aneurysm | DX | 410.xx, 441.xx |
| Acute renal failure | DX | 584.5, 584.6, 584.7, 584.8, 584.9, 669.3x |
| Adult respiratory distress syndrome | DX | 518.5, 518.81 518.82 518.84, 799.1 |
| Amniotic fluid embolism | DX | 673.1x |
| Cardiac arrest/ventricular fibrillation | DX | 427.41, 427.42, 427.5 |
| Conversion of cardiac rhythm | PR | 99.6x |
| Disseminated intravascular coagulation | DX | 286.6, 286.9, 666.3 |
| Eclampsia | DX | 642.6x |
| Heart failure or arrest during surgery or procedure | DX | 997.1 |
| Puerperal cerebrovascular disorders | DX | 430, 431, 432.x, 433.xx, 434.xx, 436, 437.x, 671.5x, 674.0x, 997.02 |
| Acute congestive heart failure or pulmonary edema | DX | 518.4, 428.1, 428.0, 428.21, 428.23, 428.31, 428.33, 428.41, 428.43 |
| Severe anesthesia complications | DX | 668.0x, 668.1x, 668.2x |
| Sepsis | DX | 038.xx, 995.91, 995.92, 670.2x |
| Shock | DX | 669.1x, 785.5x, 995.0, 995.4, 998.0x |
| Sickle cell disease with crisis | DX | 282.42, 282.62, 282.64, 282.69 |
| Air and thrombotic embolism | DX | 415.1x, 673.0x, 673.2x, 673.3x, 673.8x |
| Blood transfusion | PR | 99.0x |
| Hysterectomy | PR | 68.3x-68.9x |
| Ventilation/Temporary tracheostomy | PR | 31.1, 93.90, 96.01, 96.02, 96.03, 96.05 |

Reference: CDC. Severe maternal morbidity indicators and corresponding ICD codes during delivery hospitalizations. https://www.cdc.gov/reproductivehealth/maternalinfanthealth/smm/severe-morbidity-ICD.htm.
