# Supplementary material for: The contribution of maternal characteristics and cesarean delivery to an increasing trend of severe maternal morbidity
Source: BMC Pregnancy Childbirth. 2019 Jan 9;19:16. doi: 10.1186/s12884-018-2169-3 (PMC6327483; doi:10.1186/s12884-018-2169-3)
Supplement: Supplementary file 2 — Table S2. Comparison of characteristics for deliveries included in and excluded from the final study sample. Excluded subjects had a lower prevalence of the outcome, lower socioeconomic indicators, and delivered in earlier years. (DOCX 22 kb) [file 12884_2018_2169_MOESM2_ESM.docx]

Additional file 2: **Table S2** Comparison of characteristics for deliveries included in and excluded from the final study sample.

| **Characteristic** | **Included**  **(n = 3,556,206**  **%** | **Excluded**  **(n = 415,621)**  **%** |
| --- | --- | --- |
| Outcome |  |  |
| Severe maternal morbidity | 1.35 | 1.60 |
| Severe maternal morbidity excluding transfusion-only cases | 0.55 | 0.71 |
| Risk factors of interest – dichotomous |  |  |
| Maternal age at delivery ≥35 years | 18.3 | 19.8 |
| Pre-pregnancy obesity | 21.1 | 17.9 |
| Pre-pregnancy comorbidity | 6.8 | 6.5 |
| Cesarean delivery | 33.0 | 33.8 |
| Risk factors of interest – multiple categories |  |  |
| Maternal age at delivery (years) |  |  |
| <20 | 8.0 | 8.4 |
| 20-24 | 20.8 | 20.5 |
| 25-29 | 26.7 | 25.8 |
| 30-34 | 26.1 | 25.5 |
| 35-39 | 14.5 | 15.2 |
| ≥40 | 3.8 | 4.6 |
| Pre-pregnancy BMI group |  |  |
| Underweight | 4.0 | 4.4 |
| Normal weight | 49.0 | 52.9 |
| Overweight | 25.9 | 24.8 |
| Obesity Class 1 | 12.7 | 11.1 |
| Obesity Class 2 | 5.3 | 4.4 |
| Obesity Class 3 | 3.1 | 2.4 |
| Pre-pregnancy comorbidity |  |  |
| Pre-pregnancy hypertension | 1.8 | 1.8 |
| Pre-pregnancy diabetes mellitus | 1.1 | 1.2 |
| Asthma | 3.6 | 3.2 |
| Other comorbidity^1^ | 0.8 | 0.9 |
| Delivery method |  |  |
| Vaginal without induction | 62.5 | 62.7 |
| Vaginal with induction | 13.1 | 12.9 |
| Primary cesarean without induction | 5.6 | 4.9 |
| Primary cesarean with induction | 1.2 | 1.1 |
| Repeat cesarean | 17.6 | 18.5 |
| Sociodemographic & obstetric factors |  |  |
| Maternal education |  |  |
| Did not complete high school | 22.7 | 29.9 |
| High school graduate or GED completed | 26.1 | 27.5 |
| Some college – no degree | 24.7 | 21.6 |
| College degree or higher | 26.5 | 21.0 |
| Private health insurance | 47.6 | 42.0 |
| Maternal race/ethnicity |  |  |
| U.S.-born Hispanic/Latina | 26.4 | 20.5 |
| Foreign-born Hispanic/Latina | 25.8 | 21.7 |
| Non-Hispanic White | 25.8 | 33.6 |
| Asian/Pacific Islander | 12.5 | 14.7 |
| Non-Hispanic Black | 5.0 | 6.2 |
| Other | 4.5 | 3.3 |
| Obstetric history |  |  |
| Primiparous | 39.7 | 38.9 |
| Multiparous without prior cesarean | 43.6 | 43.6 |
| Multiparous with prior cesarean | 16.7 | 17.4 |
| Twin/multiple birth | 1.6 | 2.0 |
| Preterm birth (<37 wk gestation) | 7.7 | 9.3 |
| Placenta previa or abruption | 1.7 | 2.2 |
| Preeclampsia | 3.5 | 4.0 |

BMI, body mass index (calculated as weight in kilograms divided by height in meters squared); GED, general educational development.

^1^ Other comorbidity includes pulmonary hypertension, sickle cell disease, chronic renal disease, chronic ischemic heart disease, congenital heart disease, systemic lupus erythematosus, human immunodeficiency virus, cardiac valvular disease, and chronic congestive heart failure (26).
