# Supplementary material for: The contribution of maternal characteristics and cesarean delivery to an increasing trend of severe maternal morbidity
Source: BMC Pregnancy Childbirth. 2019 Jan 9;19:16. doi: 10.1186/s12884-018-2169-3 (PMC6327483; doi:10.1186/s12884-018-2169-3)
Supplement: Supplementary file 3 — Table S3. Observed and predicted severe maternal morbidity prevalence per 100 live births (95% confidence intervals), California, 2007–2014. The studied risk factors were related to the prevalence of SMM, but not its increase over time. (DOCX 21 kb) [file 12884_2018_2169_MOESM3_ESM.docx]

Additional file 3: **Table S3** Observed and predicted severe maternal morbidity prevalence per 100 live births (95% confidence intervals), California, 2007-2014

| **Risk factor set to zero prevalence** | **2007** | **2008** | **2009** | **2010** | **2011** | **2012** | **2013** | **2014** |
| --- | --- | --- | --- | --- | --- | --- | --- | --- |
| None (observed) | 1.02 | 1.11 | 1.26 | 1.31 | 1.37 | 1.49 | 1.56 | 1.69 |
| Advanced maternal age | 0.93  (0.40, 1.93) | 1.02  (0.44, 2.03) | 1.17  (0.56, 2.26) | 1.22  (0.55, 2.43) | 1.28  (0.58, 2.42) | 1.39  (0.68, 2.76) | 1.44  (0.65, 2.84) | 1.58  (0.81, 2.96) |
| Pre-pregnancy obesity | 1.03  (0.43, 2.21) | 1.11  (0.47, 2.47) | 1.26  (0.60, 2.63) | 1.32  (0.58, 2.83) | 1.37  (0.61, 2.79) | 1.49  (0.70, 3.16) | 1.59  (0.68, 3.37) | 1.69  (0.83, 3.22) |
| Pre-pregnancy comorbidity | 0.94  (0.39, 1.95) | 1.02  (0.43, 2.16) | 1.16  (0.55, 2.33) | 1.22  (0.54, 2.53) | 1.27  (0.56, 2.49) | 1.38  (0.66, 2.82) | 1.43  (0.62, 2.95) | 1.56  (0.78, 2.89) |
| All 3 pre-pregnancy risk factors | 0.89  (0.37, 1.73) | 0.96  (0.42, 1.81) | 1.11  (0.53, 2.00) | 1.15  (0.52, 2.21) | 1.21  (0.55, 2.16) | 1.32  (0.65, 2.48) | 1.39  (0.62, 2.58) | 1.49  (0.77, 2.64) |
| Cesarean delivery | 0.62  (0.27, 2.13) | 0.68  (0.30, 2.23) | 0.78  (0.37, 2.62) | 0.84  (0.40, 2.86) | 0.83  (0.40, 2.76) | 0.94  (0.48, 3.08) | 1.00  (0.46, 3.28) | 1.12  (0.59, 3.63) |
| All 4 risk factors | 0.59  (0.27, 1.96) | 0.64  (0.30, 2.03) | 0.75  (0.38, 2.45) | 0.80  (0.40, 2.66) | 0.81  (0.41, 2.56) | 0.91  (0.50, 2.89) | 0.97  (0.49, 3.07) | 1.08  (0.62, 3.37) |
