# Supplementary material for: The contribution of maternal characteristics and cesarean delivery to an increasing trend of severe maternal morbidity
Source: BMC Pregnancy Childbirth. 2019 Jan 9;19:16. doi: 10.1186/s12884-018-2169-3 (PMC6327483; doi:10.1186/s12884-018-2169-3)

**Additional file 4: Figure S1. Annual prevalence of severe maternal morbidity excluding blood transfusion-only cases, California, 2007-2014.**


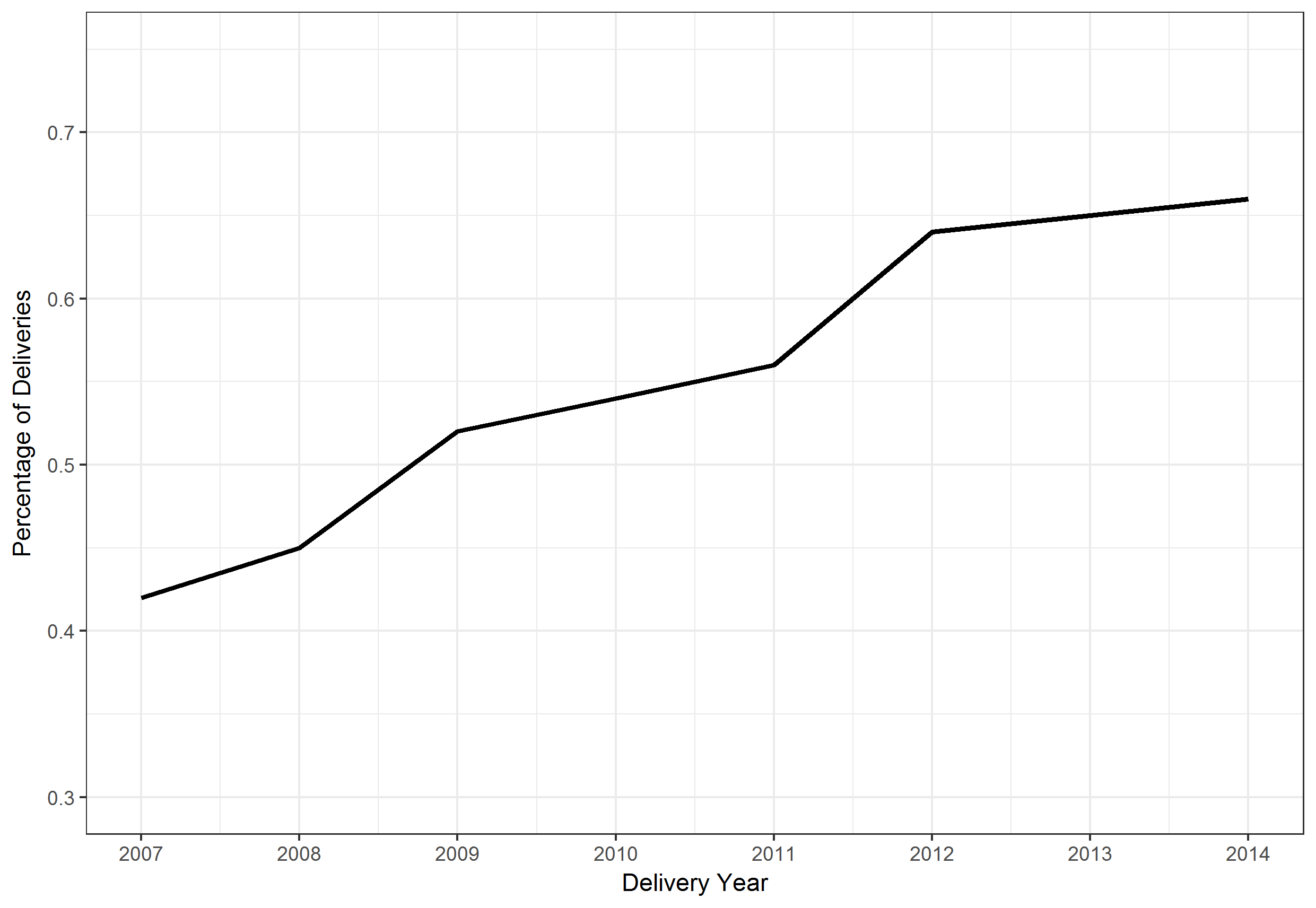

Supplement: Supplementary file 4 — Figure S1. Annual prevalence of severe maternal morbidity excluding blood transfusion-only cases, California, 2007–2014. The prevalence increased from 2007 to 2012. (DOCX 66 kb) [file 12884_2018_2169_MOESM4_ESM.docx]
